# Investigation into Cardiac Myhc-α 334–352-Specific TCR Transgenic Mice Reveals a Role for Cytotoxic CD4 T Cells in the Development of Cardiac Autoimmunity

**DOI:** 10.3390/cells13030234

**Published:** 2024-01-26

**Authors:** Meghna Sur, Mahima T. Rasquinha, Kiruthiga Mone, Chandirasegaran Massilamany, Ninaad Lasrado, Channabasavaiah Gurumurthy, Raymond A. Sobel, Jay Reddy

**Affiliations:** 1 School of Veterinary Medicine and Biomedical Sciences, University of Nebraska-Lincoln, Lincoln, NE 68588, USA; msur2@huskers.unl.edu (M.S.); mrasquinha2@huskers.unl.edu (M.T.R.); kmone2@huskers.unl.edu (K.M.); mchandirasegaran@gmail.com (C.M.); nlasrado@bidmc.harvard.edu (N.L.); 2 CRISPR Therapeutics, Boston, MA 02127, USA; 3 Center for Virology and Vaccine Research, Harvard Medical School, Boston, MA 02115, USA; 4 Department of Genetics, Cell Biology and Anatomy, University of Nebraska Medical Center, Omaha, NE 68198, USA; cgurumurthy@unmc.edu; 5 Department of Pathology, Stanford University, Stanford, CA 94305, USA; raysobel@stanford.edu

**Keywords:** TCR transgenic mice, cardiac myosin-α 334–352, myocarditis, inflammatory cardiomyopathy

## Abstract

Myocarditis is one of the major causes of heart failure in children and young adults and can lead to dilated cardiomyopathy. Lymphocytic myocarditis could result from autoreactive CD4^+^ and CD8^+^ T cells, but defining antigen specificity in disease pathogenesis is challenging. To address this issue, we generated T cell receptor (TCR) transgenic (Tg) C57BL/6J mice specific to cardiac myosin heavy chain (Myhc)-α 334–352 and found that Myhc-α-specific TCRs were expressed in both CD4^+^ and CD8^+^ T cells. To investigate if the phenotype is more pronounced in a myocarditis-susceptible genetic background, we backcrossed with A/J mice. At the fourth generation of backcrossing, we observed that Tg T cells from naïve mice responded to Myhc-α 334–352, as evaluated by proliferation assay and carboxyfluorescein succinimidyl ester staining. The T cell responses included significant production of mainly pro-inflammatory cytokines, namely interferon (IFN)-γ, interleukin-17, and granulocyte macrophage-colony stimulating factor. While the naïve Tg mice had isolated myocardial lesions, immunization with Myhc-α 334–352 led to mild myocarditis, suggesting that further backcrossing to increase the percentage of A/J genome close to 99.99% might show a more severe disease phenotype. Further investigations led us to note that CD4^+^ T cells displayed the phenotype of cytotoxic T cells (CTLs) akin to those of conventional CD8^+^ CTLs, as determined by the expression of CD107a, IFN-γ, granzyme B natural killer cell receptor (NKG)2A, NKG2D, cytotoxic and regulatory T cell molecules, and eomesodermin. Taken together, the transgenic system described in this report may be a helpful tool to distinguish the roles of cytotoxic cardiac antigen-specific CD4^+^ T cells vs. those of CD8^+^ T cells in the pathogenesis of myocarditis.

## 1. Introduction

Inflammatory heart disease comprises a group of disorders that affect different layers of the heart. These include pericarditis (inflammation of the outer membranous sac), myocarditis (inflammation of the heart muscle), and endocarditis (inflammation of the inner lining of the heart, heart valves, and/or coronary arteries) [1,2]. Myocarditis, in particular, may involve cardiac myocytes, the interstitial spaces, or the vascular background of the heart and pericardium. Some affected patients show clinical manifestations of disease while others remain asymptomatic, but histopathologic changes can be detected in those affected [3]. The disease, generally regarded as an underdiagnosed cause of acute heart failure, may lead to sudden death or dilated cardiomyopathy in young adults [4]. Globally, myocarditis prevalence is estimated in the range of 10.2 to 105.6 per 100,000, with an occurrence of ~1.8 million cases per year as of 2017, a strong indicator of the importance of the disease [5].

Myocarditis can result from a plethora of infectious and non-infectious causes, including side effects resulting from the use of checkpoint inhibitor therapies and vaccines [6,7,8,9]. Of the many types of myocarditis defined by histological characteristics [10], lymphocytic myocarditis is characterized by variable numbers of lymphocytes and macrophages, but antibody-mediated injury can also be expected [11,12], implying that autoimmunity may be an underlying mechanism. 

Unlike autoinflammatory diseases that result from the direct involvement of innate immune cells such as neutrophils and macrophages, autoimmune diseases are primarily mediated by adaptive immune cells, T cells, and B cells. While both types of cells can mediate autoimmune reactions in their own pathogenic pathways, T cells—especially CD4^+^ helper T (Th) cells—occupy a central role because cytokines produced by Th cells are critical in helping B cells produce antibodies to protein antigens. The majority of autoantibodies target protein antigens of self-tissues in various organ-specific autoimmune diseases, and myocarditis is no exception [13]. While Th1 and Th17 cells may cooperatively mediate myocarditis progression to inflammatory dilated cardiomyopathy (iDCM), interferon (IFN)-γ-producing Th1 cells may have a disease-protective role [14]. Likewise, CD4^+^ regulatory T (Treg) cells may attenuate cardiac inflammation and prevent development of iDCM [15]. Nonetheless, because of the plasticity of the functionalities of Th1, Th17, and Treg cells [16,17], it is critical to dissect their role in cardiac diseases at the level of antigen specificity. Similarly, CD8^+^ T cells may contribute to cardiac damage through cytotoxicity, but their role has not been well studied. Various adjuvant models of myocarditis have been developed to address the role of T cells, but the challenge is being able to define their antigen specificity. This limitation can be abated with the development of T cell receptor (TCR) transgenic (Tg) mice specific to cardiac antigens. We recently reported the generation of TCR Tg mice specific to cardiac myosin heavy chain (Myhc)-α 334–352 on a myocarditis-resistant C57BL/6J background [18]. In this report, we describe the characterization of Tg mice backcrossed with myocarditis-susceptible A/J mice for four generations. We also present evidence that the cytotoxic functions of CD4^+^ and CD8^+^ T cells expressing Myhc-α 334–352-specific TCRs are comparable in the causation of myocarditis in these mice.

## 2. Materials and Methods

### 2.1. Generation and Screening of TCR Transgenic (Tg) Mice

We recently reported the generation and characterization of TCR Tg mice for Myhc-α 334–352 under a C57BL/6J background [18], and we backcrossed Tg mice with A/J mice for four generations, hereafter termed A/J Tg mice. They were genotyped by quantitative PCR (qPCR) using genomic DNA extracted from mouse tails, as previously described [18]. For backcrossing, male TCR Tg mice (C57BL/6J background) were bred with A/J female mice obtained from Jackson Laboratory (Bar Harbor, ME, USA). All animals were maintained according to the institutional guidelines of the University of Nebraska-Lincoln, Lincoln, NE, USA. We have donated A/J Tg mice for cryopreservation at the Mutant Mouse Resource and Research Center (St. Louis, MO, USA) (Tg mice expressing TCR-α chain alone: MMRRC:068226-MU; Tg mice expressing TCR-β chain alone: MMRRC:069589-MU; and Tg mice expressing both TCR-α and β chains: MMRRC:069590-MU).

### 2.2. Peptide Synthesis

Myhc-α 334–352 (DSAFDVLSFTAEEKAGVYK) and RNase 43–56 (VNTFVHESLADVQA) was synthesized on 9-fluorenylmethyloxycarbonyl chemistry (Genscript, Piscataway, NJ, USA). All peptides were high-performance liquid chromatography (HPLC)-purified, and more than 90% purity was confirmed by HPLC and mass spectroscopy. The peptides were dissolved in 1× phosphate-buffered saline, aliquoted, and stored at −20 °C.

### 2.3. Immunophenotyping by Flow Cytometry

#### 2.3.1. Surface Staining

Splenocytes and thymocytes were analyzed ex vivo for various markers using the following antibodies, with their clone identifiers shown in parentheses: CD3 (17A2); CD4 (GK1.5); CD8 (YTS156.7.7); CD44 (IM7); CD62L (MEL-14) (BioLegend, San Diego, CA, USA); and TCR Vβ4 (KT4) (BD Biosciences, Franklin Lakes, NJ, USA). For surface marker staining, cells were stained with antibodies on ice for 15 min, and after washing, cells were acquired by flow cytometry (FACS Calibur™, BD Biosciences or CytoFLEX, Beckman Coulter Life Sciences, Indianapolis, IN, USA) and analyzed using FlowJo software v10.9 (Tree Star, Ashland, OR, USA).

#### 2.3.2. Intracellular Staining

##### Transcription Factors

After staining the cells with antibodies for CD4 (GK1.5) and CD8 (YTS156.7.7), anti-forkhead box P3 (FoxP3, clone 150D) and anti-eomesodermin (EOMES, clone W17001A) (BioLegend) were used for intranuclear staining. Cells were washed, fixed, and permeabilized in the dark at room temperature (RT) using True-Nuclear™ Fix buffer (BioLegend). After 1 h, the cells were washed three times, followed by staining with anti-FoxP3 or anti-EOMES at RT for 30 min. The cells were washed and after acquisition by flow cytometry, cells positive for FoxP3 and EOMES in CD4^+^ and CD8^+^ subsets were analyzed using FlowJo.

##### Cytokines

Single cell suspensions, termed lymphocytes, were harvested from spleens and draining lymph nodes. Cells at a density of 5 × 10^6^ cells/mL were stimulated with Myhc-α 334–352 or RNase 43–56 (50 µg/mL) in the growth medium. After two days, interleukin (IL)-2 media was added. On day 4, the cells were stimulated briefly with a cell activation cocktail (BioLegend) for ~5 h. Cells were washed and stained with antibodies for CD4 (GK1.5), CD8 (YTS156.7.7), and 7-aminoactinomycin D (7-AAD) (BioLegend). After washing, cells were fixed and permeabilized in the dark at RT using Cyto-Fast™ Fix/Perm buffer (BioLegend). After 20 min, the cells were washed and stained for 20 min with the following cytokine antibodies, with clones indicated in parentheses: IL-2 (JES6-5H4); IFN-γ (XMG1.2); IL-4 (11B11); IL-10 (JES5-16E3); IL-17A (TC11-18H10.1); and granulocyte-macrophage colony-stimulating factor (GM-CSF) (MP1-22F9). Cells were incubated in the dark at RT for 20 min and washed twice. After acquiring cells by flow cytometry, cytokine^+^ CD4^+^ and CD8^+^ T cells in viable populations (7-AAD^−^) were analyzed using FlowJo [19,20]. In some experiments, cytokine-producing CD4 and CD8 T cells were analyzed in relation to CD62L and CD44 expression in the respective populations.

#### 2.3.3. Cytotoxic T Cell (CTL) Markers

Lymphocytes prepared from Tg mice were stimulated with Myhc-α 334–352 or RNase 43–56 (50 µg/mL) in RPMI-1640 medium supplemented with growth factors and antibiotics, as previously described [18]. After two days, IL-2 media was added. On day 4, the cells were stimulated briefly with monensin (BioLegend) for 5 h and stained with the following antibodies, and their clones are shown in parentheses: CD4 (GK1.5); CD8 (YTS156.7.7); CD107a (1D4B); Natural Killer Cell Receptor (NKG)2A (705829); NKG2D (CX5); and Cytotoxic and Regulatory T Cell Molecule (CRTAM) (11-5/CRTAM). Intracellular staining was performed for granzyme B (GrB) (QA16A.02) and IFN-γ (XMG1.2), and intranuclear nuclear staining was performed for EOMES (W17001A), as described above. All antibodies except NKG2A (R&D Systems, Minneapolis, MN, USA) were procured from BioLegend.

### 2.4. RNA Extraction and Quantitative Polymerase Chain Reaction

Approximately 20–30 mg of heart, thymus, and spleen tissue harvested from various strains of mice were snap-frozen in liquid nitrogen and stored at −80 °C. For RNA isolation, tissues were transferred to lysis buffer containing β-mercaptoethanol and homogenized using the FastPrep-24™ system, according to the manufacturer’s recommendations (Lysing Matrix D 1.4-mm ceramic beads; MP Biomedicals, Irvine, CA, USA). RNA was extracted using the RNA Mini Kit (PureLink™, Thermo Fisher Scientific, Waltham, MA, USA) and eluted in RNase-free water. The isolated RNA samples were treated with deoxyribonuclease (DNase) I and quantified using the NanoDrop ND-1000 spectrophotometer (Thermo Fisher Scientific). In a single-step reaction, 50 ng of RNA was reverse-transcribed, and qPCR was performed using the iTaq™ Universal SYBR^®^ Green One-Step Kit. Real-time qPCR analysis was performed using sequence-specific primers for mouse Myhc-α (5′-GCTACACTCTTCTCTACC-3′ and 5′-CATAGAGAATGCGGTTGG-3′) and for glyceraldehyde-3-phosphate dehydrogenase (GAPDH) (5′CTCCCACTCTTCCACCTTCG-3′ and 5′GCCTCTCTTGCTCAGTGTCC-3′). The CFX96 Touch Real-time PCR detection system (BioRad, Hercules, CA, USA) was used for thermocycling, and the relative normalized gene expression of mouse cardiac myosin was analyzed using the 2^−(ΔΔCt)^ method [21] in BioRad CFX Maestro 1.0 software.

### 2.5. Proliferation Assay

Lymphocytes were stimulated with Myhc-α 334–352, RNase 43–56 (0–100 µg/mL), anti-CD3 (clone 145-2C11, 2.5 µg/mL), or mouse cardiac myosin protein (0–100 µg/mL) at a concentration of 2.5 × 10^6^ cells/mL for two days in RPMI-1640 medium. Cells were pulsed with tritiated [3H] thymidine and after 16 h, the responses of proliferating cells were measured using a Wallac liquid scintillation counter as counts per minute (CPM) (Perkin Elmer, Waltham, MA, USA) [18,19].

### 2.6. Purification of Mouse Cardiac Myosin

Cardiac myosin protein was purified as previously described with a few modifications [22]. Approximately 75 mouse hearts were freshly harvested and stored at −80 °C until further use. Frozen hearts were thawed on ice and rinsed in ice-cold phosphate-buffered saline (1× PBS) overnight. Hearts were finely chopped and minced using the POLYTRON^®^ PT 1200 E (Kinematica AG, Malters, Switzerland) in modified Hasselbach–Schneider solution. The homogenate was extracted by stirring and centrifuged at 12,000× *g* to remove muscle residue, followed by another round of centrifugation at 140,000× *g*. The supernatant was precipitated in ice-cold water overnight, and the redissolved precipitate was centrifuged at 43,000× *g* to remove actin. The supernatant was once again precipitated in ice-cold water overnight, and the redissolved precipitate was centrifuged at 43,000× *g* to remove actomyosin. Myosin from the supernatant was precipitated in ice-cold water overnight and redissolved in 50% glycerol buffer containing 0.6 M KCl and 10 mM KH_2_PO_4_ at pH 7. The protein concentration was measured using the bicinchoninic protein assay and confirmed on polyacrylamide gel electrophoresis.

### 2.7. Cytokine Bead Array Analysis

Lymphocytes were obtained from A/J Tg mice that were immunized with Myhc-α 334–352, unimmunized naïve animals, or their littermates. Where shown, cells were restimulated with anti-CD3 (clone 145-2C11, 2.5 µg/mL), Myhc-α 334–352, or RNase 43–56 (10–100 µg/mL), and the supernatants from cell cultures were collected. Cytokine analysis was performed using the Murine Th Cytokine Panel (12-plex; BioLegend), consisting of IFN-γ, IL-2, IL-4, IL-5, IL-13, IL-9, IL-17A, IL-17F, IL-22, IL-6, tumor necrosis factor (TNF)-α, and IL-10. The lyophilized mouse cytokine standard mix was serially diluted to obtain the standard curve. After adding the capture beads/cytokine antibody conjugates, detection antibodies were added to the standards and samples, followed by streptavidin–phycoerythrin reagents. After acquisition of beads by flow cytometry, concentrations of cytokines were analyzed using the LEGENDplex™ software suite, Version 2023-02-15 (BioLegend) [18,23].

### 2.8. Carboxyfluorescein Diacetate Succinimidyl Ester (CFSE) Assay

The CFSE assay was performed by adopting the procedure previously described [24]. CFSE (BioLegend) was reconstituted in anhydrous dimethyl sulfoxide to a stock concentration of 5 mM. Lymphocytes (5 × 10^6^) were resuspended in 1 mL of RPMI containing 5% heat-inactivated (HI)-FBS. The working concentration of CFSE was prepared by adding 1.1 µL of 5 mM stock CFSE to 110 µL of PBS and applying it to a non-wetted region of a 15 mL conical tube, laying it horizontally to achieve a final concentration of 5 µM. The tubes were capped, inverted, and immediately vortexed to achieve homogeneous mixing of the cell suspension. Cells were incubated at RT for 5 min and washed with 1× buffer (HI-FBS/RT PBS) three times by centrifuging at 300× *g* for 5 min at 20 °C. The labeled lymphocytes were resuspended in RPMI-1640 medium and cells were stimulated with or without Myhc-α 334–352 or RNase 43–56 (50 µg/mL) for 3 days after IL-2 medium was added on day 2. The labeled unstimulated cells, as well as labeled and unlabeled cells stimulated with anti-CD3, were used as controls. On day 4, cells were stained with antibodies for CD4, CD8, and 7-AAD, and after cells were acquired by flow cytometry, the data were analyzed by FlowJo software v10.9.

### 2.9. Immunization

Immunization involved Myhc-α 334–352 emulsified in complete Freund’s adjuvant (CFA) supplemented with an extract of *Mycobacterium tuberculosis* H37RA (Difco Laboratories, Detroit, MI, USA) to 5 mg/mL concentration [18]. The peptide emulsion was subcutaneously injected (100 µg/mouse) into the shoulder and hip regions (left and right) in split doses. In addition, animals received pertussis toxin (List Biological Laboratories, Campbell, CA, USA), intraperitoneally (100 ng/mouse) on days 0 and 2 after the first immunization.

### 2.10. Histopathology

Hearts were fixed by immersion in 10% phosphate-buffered formalin and routinely processed for histology. Three cross-sections of heart were obtained with a thickness of 5 µm each, and they were stained with hematoxylin and eosin (H and E). Sections were examined by a board-certified pathologist for inflammatory changes and analyzed blinded to treatment for severity based on inflammatory changes [25].

### 2.11. Statistical Analysis

Statistical analysis was performed and graphs were prepared using GraphPad Prism software v8.0 (GraphPad Software, Inc. La Jolla, CA, USA). The Shapiro–Wilk test was used to test for normality for all datasets. Datasets pertaining to immunophenotyping, CFSE, proliferative response, and cytokine bead array were analyzed by Student’s *t*-test or two-way ANOVA. A *p*-value ≤ 0.05 was considered statistically significant.

## 3. Results and Discussion

In this report, we describe the characterization of Myhc-α 334–352-specific TCR A/J Tg mice and their phenotype in developing cardiac autoimmunity. We recently reported the generation of Tg mice on a myocarditis-resistant C57BL/6J background and described their phenotypic characterization [18]. While T cells from naïve Tg mice did not respond to Myhc-α 334–352, both CD4^+^ and CD8^+^ T cells from immunized mice responded, suggesting that in vivo priming was necessary to break tolerance in myocarditis-resistant mice. Additionally, C57BL/6J Tg mice immunized with Myhc-α 334–352 developed mild myocarditis despite producing proinflammatory cytokines, indicating that the genetic elements of C57BL/6J mice may contribute to disease resistance. We then backcrossed Tg mice with myocarditis-susceptible A/J mice for up to four generations, generating mice containing more than 90% A/J genome, and characterized their immune and disease phenotypes.

**Figure 1:**
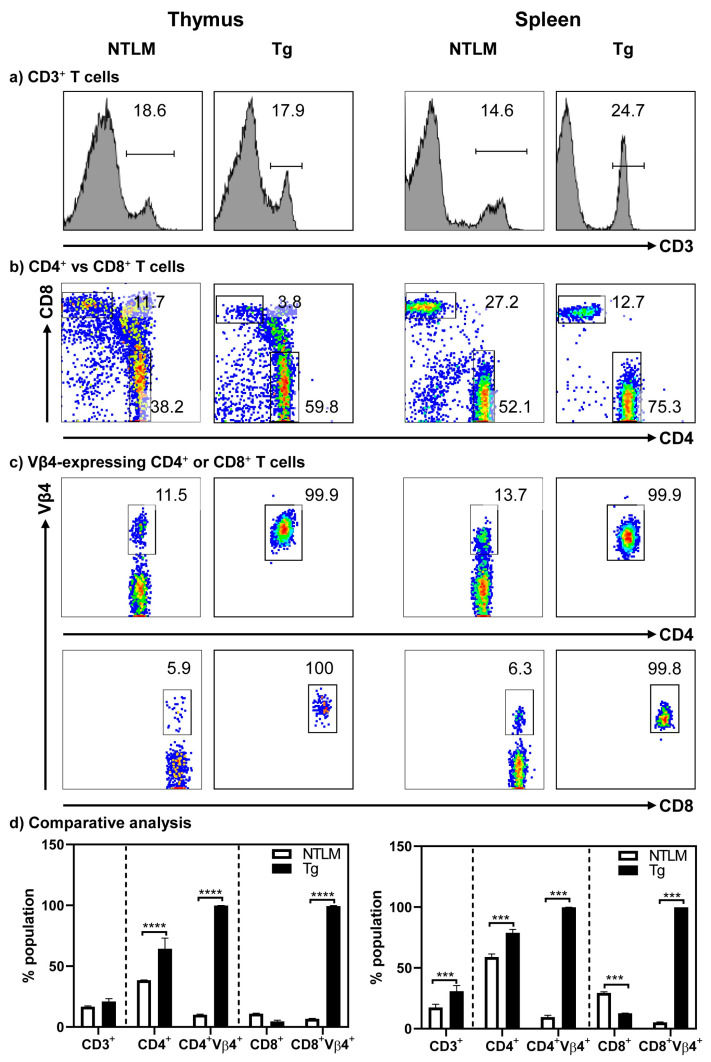
Tg mice express Myhc-α 334–352-specific TCRs on both CD4^+^ and CD8^+^ T cells. Thymocytes and splenocytes were prepared from NTLM and Tg mice, and the cells were stained with antibodies for CD3, CD4, CD8, and TCR vβ4. Their corresponding cell populations were analyzed by flow cytometry, as shown in left and right panels, respectively. While panels (**a**–**c**) respectively represent the flow cytometric plots of the subsets of cells positive for anti-CD3, CD4, and CD8, and TCR vβ4 in CD4^+^ and CD8^+^ T cells, panel (**d**) indicates the comparative analysis of the above populations. Mean ± SEM values obtained from three to four mice in each group are shown. *** *p* ≤ 0.001 and **** *p* ≤ 0.0001.

Characterization of T cell subsets revealed that the CD3^+^ cells were similar in the thymocytes of both A/J Tg mice and their non-transgenic littermates (NTLMs). In the periphery, however, the abundance of CD3^+^ T cells was higher in the splenocytes of Tg mice as compared to NTLMs (30.9 ± 4.6% vs. 17.4 ± 2.7%, *p* ≤ 0.001) (Figure 1a,d). A similar comparison of CD4^+^ and CD8^+^ T cells revealed skewing of CD4^+^ T cells in both thymocytes (64.4 ± 8.7% vs. 38.45 ± 0.3%, *p* ≤ 0.001) and splenocytes (78.8 ± 2.9% vs. 58.9 ± 2.5%, *p* ≤ 0.001) in Tg mice as compared to NTLMs. Correspondingly, CD8^+^ T cell populations, especially in the spleen, were reduced (12.7 ± 0.1% vs. 29.4 ± 0.9%, *p* ≤ 0.0001) (Figure 1b,d). Such skewed CD4 to CD8 ratios have been reported in other transgenic models, and asymmetric cell death of CD4^+^ and CD8^+^ thymocytes during development may be an underlying mechanism [26,27]. After their export, the skewed ratio may continue to be maintained in the periphery. Additionally, the degree of CD4 to CD8 skewing may also differ between mouse strains [28]. Importantly, 100% of CD4^+^ or CD8^+^ T cells in both thymocytes and splenocytes expressed the transgenes, as analyzed by staining with TCR-vβ4 antibody (Figure 1c,d). Furthermore, by analyzing memory T cell markers (CD62L and CD44), we noted that Tg mice had relatively higher numbers of CD62L^+^ CD4^+^ cells (79.4 ± 1.3% vs. 53.37 ± 0.9%, *p* ≤ 0.001) and CD8^+^ T cells (53.8 ± 3.8% vs. 44.5 ± 1.3%, *p* ≤ 0.05), whereas the number of CD44+ cells was low (8.9 ± 1.3% vs. 28.37 ± 0.9%, *p* ≤ 0.001) in the CD4^+^ subset as compared to NTLMs (Figure S1). The number of FoxP3-expressing CD4^+^ T cells, if any, was low in Tg mice relative to their littermates (8.9 ± 0.6% vs. 17.7 ± 2.1%, *p* ≤ 0.01) (Figure S1). Taken together, the data suggested that Tg T cells might have escaped thymic deletion.

To address the above, we analyzed the expression of cardiac myosin in the lymphoid organs (thymus and spleen) harvested from wild-type A/J and C57BL/6J mice, as well as A/J Tg mice and their littermates, by qPCR using cardiac myosin-specific primers spanning the genomic region encompassing Myhc-α 334–352. By using heart tissues from wild-type A/J and C57BL/6J as positive controls, we noted that myosin mRNA expression was detected in the hearts of both strains (Figure S2). Similar analysis led us to note the lack of myosin expression in the spleen, whereas thymus tissue had negligible levels, if any, of myosin expression in all mouse strains (Figure S2). Of note, thymic education of T cells involving positive and negative selection events depends on the recognition of self-antigens in the context of major histocompatibility complex (MHC) molecules associated with weak and strong interactions, respectively [29,30]. Furthermore, the lack or low expression of self-antigens in the thymus leads to the export of self-reactive T cells to the periphery [31,32]. As the thymus from A/J Tg mice had only negligible expression of myosin, T cells might have escaped thymic selection. Consistent with this observation, it has been previously reported lack of myosin expression in BALB/c mice and non-obese diabetic mice might have led to the escape of myosin-reactive T cells to the periphery [33,34], and transgenic expression of myosin in the thymus led to tolerance [34]. Taken together, it appears that the majority of commonly used mouse strains appear not to express myosin in their thymus, and characterization of myosin-reactive T cells in different genetic backgrounds may offer new insight into the tolerance mechanisms.

**Figure 2:**
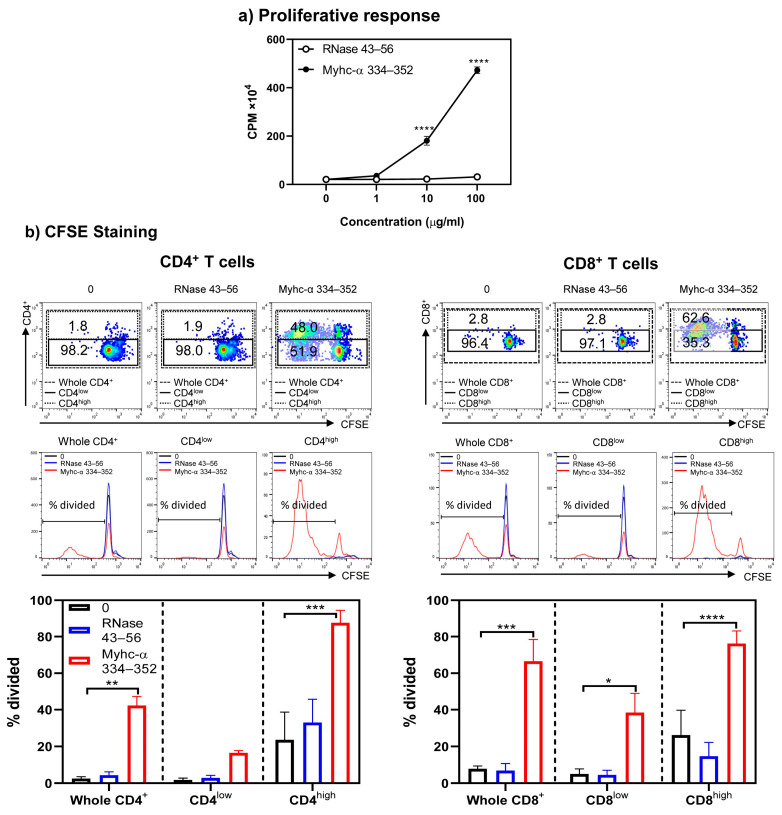
Both CD4^+^ and CD8^+^ T cells from naïve Tg mice respond to Myhc-α 334–352 and their responses are comparable. (**a**) Proliferative response. Lymphocytes prepared from naïve Tg mice were stimulated with Myhc-α 334–352 or RNase 43–56 (control) for two days, and after pulsing with [3H] thymidine for 16 h, proliferative responses were measured as CPM. (**b**) CFSE staining. Lymphocytes were stained with CFSE prior to stimulation with or without Myhc-α 334–352 or RNase 43–56 (50 µg/mL). IL-2 medium was added on day 2, and cells harvested on day 4 were stained with anti-CD4, anti-CD8, and 7-AAD. The top, middle, and bottom panels, respectively, represent the flow cytometric plots of cells positive for CD4 and CD8 in relation to CFSE; histogram plots of overlays between medium, RNase 43–56, and Myhc-α 334–352 treatments; and bar diagrams of whole CD4^+^ or CD8^+^ T cells or their low- and high-expressing cells. Mean ± SEM values are obtained from three mice in each group. * *p* ≤ 0.05, ** *p* ≤ 0.01, *** *p* ≤ 0.001, and **** *p* ≤ 0.0001.

Next, to determine the functionalities of Tg T cells, we initially evaluated their response to Myhc-α 334–352 using the [3H] thymidine incorporation assay. As shown in Figure 2, lymphocytes obtained from naïve A/J Tg mice responded to Myhc-α 334–352 dose dependently (up to 23-fold, *p* ≤ 0.0001) (Figure 2a). The responses were specific, since the cells did not respond to RNase 43–56 (control), which also binds the MHC class II/IA^k^ molecule [29]. However, by testing the response of A/J Tg T cells to the whole cardiac myosin protein, we noted that T cells did not react to the protein (Figure S3). Myhc-α 334–352, which binds to the IA^k^ molecule, is the only immunogenic peptide identified in the whole myosin protein in A/J mice [30,31,32]. Although lack of response to the whole myosin protein was unexpected, reports indicate the existence of two types of CD4^+^ T cells, Type A and Type B, based on their reactivity to peptides and proteins [33,34,35]. Type A T cells follow the conventional recognition of peptides presented by antigen-presenting cells (APCs) pulsed with both whole protein and peptides derived from the protein. Type B T cells react in a non-conventional way and are activated by APCs loaded with soluble peptides but not by APCs pulsed with the parent protein of these peptides [33,34,35]. The T cell response noted in our Tg model was similar to that of Type B T cells, which may have a role in developing cardiac autoimmunity. Next, to determine differential responses, if any, between CD4^+^ and CD8^+^ T cells, we performed CFSE staining on Tg T cells stimulated with or without Myhc-α 334–352 or RNase 43–56, including 7-AAD, to differentiate between live and dead populations. We noted that CD4 and CD8 co-receptors were upregulated only in cultures stimulated with Myhc-α 334–352 but not in controls (medium or RNase 43–56), and the proliferating cells, as analyzed by dilution of CFSE, were found mainly in high populations of CD4^+^ or CD8^+^ cells (Figure 2b, top panel). We next analyzed the percentage of divided cells in the whole CD4^+^ or CD8^+^ T subsets and in the low and high subsets of CD4^+^ or CD8^+^ populations, leading us to note that the majority of CD4^+^ and CD8^+^ T cells were divided mainly in their high subsets (Figure 2b, middle and bottom panels). Such cell divisions were lacking in cells cultured in medium alone or in cells stimulated with RNase 43–56. Taken together, in response to Myhc-α 334–352, Tg T cells that exhibited increased expression of CD4 and CD8 corresponded to cell division, as indicated by CFSE staining, implying that low and high CD4^+^ and CD8^+^ T cell populations reflect unactivated and activated populations, respectively. Detection of CD4^+^ and CD8+ T cell reactivity to Myhc-α 334–352 was not surprising since this peptide contains the determinants for both cell types [30,31]. Similar reactivity of CD4^+^ and CD8^+^ T cells has been reported for another autoantigen, myelin oligodendrocyte glycoprotein 35–55 [36,37]. The availability of such Tg models may facilitate investigating the roles of both CD4^+^ and CD8^+^ T cells within a single transgenic system.

**Figure 3:**
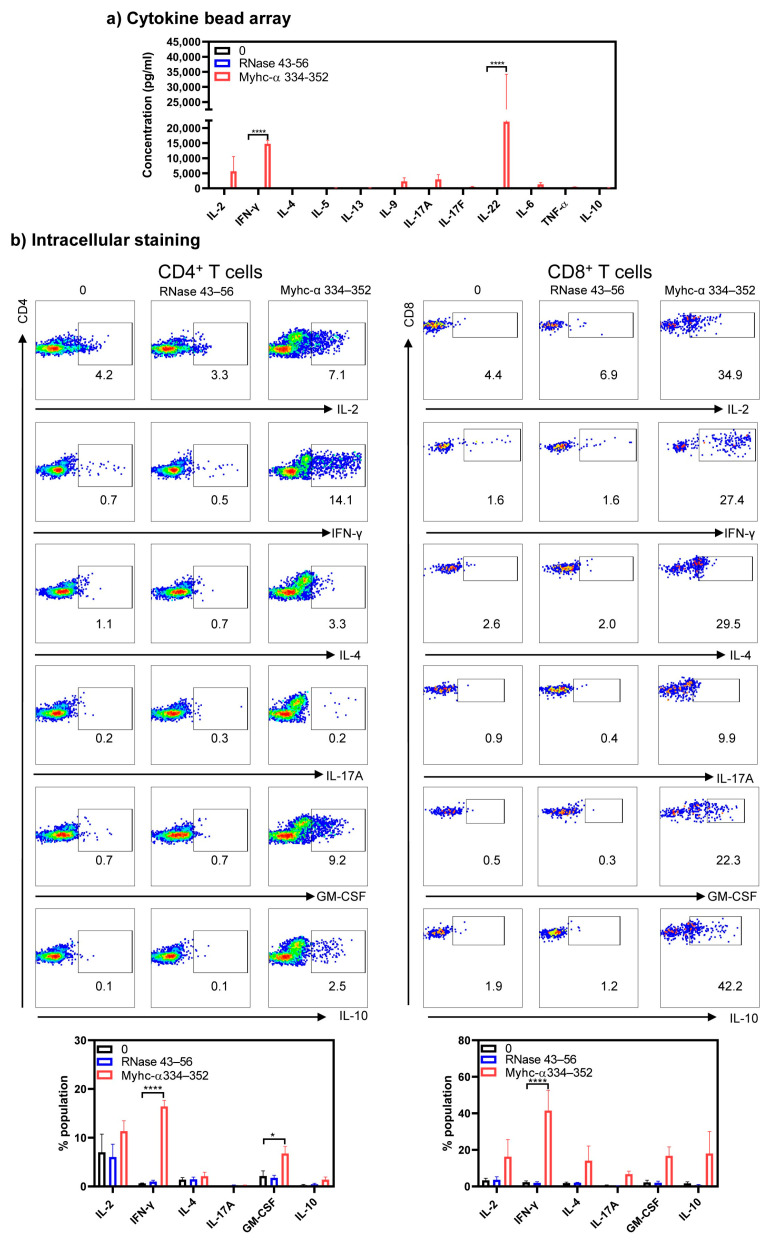
Naïve Tg T cells produce cytokines representing various T helper subsets. (**a**) Cytokine bead array analysis. Lymphocytes from naïve Tg mice were stimulated with or without Myhc-α 334–352 or RNase 43–56 (control) (50 µg/mL) for two days, and supernatants were analyzed for the indicated cytokines using the LEGENDplex Murine Th Cytokine Panel (12-plex; BioLegend), as described in the Methods section. (**b**) Intracellular staining. Lymphocytes were stimulated as above for two days, and IL-2 medium was then added. Cells harvested on day 4 were stained with anti-CD4, anti-CD8, and 7-AAD; after fixing and permeabilizing, cells were stained with cytokine antibodies, and cytokine-positive cells within CD4 or CD8 subsets were analyzed by flow cytometry. Mean ± SEM values are obtained from three mice in each group. * *p* ≤ 0.05 and **** *p* ≤ 0.0001.

We next determined cytokine profiles in response to Myhc-α 334–352 by cytokine bead array analysis and intracellular staining. Cytokine bead array analyses detected Th1 (IL-2 and IFN-γ) and Th17 (IL-17A, IL-17F, and IL-22), in addition to IL-9, IL-6, and TNF-α in response to Myhc-α 334–352. By comparing these profiles between treatments, we noted significant elevations in IFN-γ (14,766.4 ± 1160.6 pg/mL, *p* ≤ 0.0001) and IL-22 (22,134.6 ± 12,067.7 pg/mL, *p* ≤ 0.001) in response to Myhc-α 334–352 (Figure 3a). Of note, lymphocytes treated with a polyclonal activator (anti-CD3) produced mainly IFN-γ (3100.9 ± 1087.8 pg/mL, *p* ≤ 0.001) (Figure S4, top panel). Similar patterns were noted with the lymphocytes obtained from NTLMs in response anti-CD3 with IFN-γ (12,000.0 ± 0.0 pg/mL, *p* ≤ 0.0001) (Figure S4, top panel), and to some degree, IL-17 (1013.6 ± 545.5 pg/mL, *p* ≤ 0.05) being produced more than others (Figure S4, bottom panel). Thus, cytokines produced in response to Myhc-α 334–352 may have pathologic significance. Furthermore, by analyzing the frequencies of cytokine-producing cells, we noted significant increases in the frequencies of IFN-γ-producing CD4^+^ (16.4 ± 1.3%, *p* ≤ 0.0001) and CD8^+^ (41.5 ± 11.1%, *p* ≤ 0.0001) as compared to controls (Figure 3b, top and bottom panels). While the frequency of GM-CSF-producing cells was also significantly high in the CD4^+^ subset (6.7 ± 1.5%, *p* ≤ 0.05), similar trends existed with CD8^+^ T cells (Figure 3b, top and bottom panels). Of note, by evaluating the memory T cell populations, we noted that CD4^+^ or CD8^+^ T cells cultured in the presence of Myhc-α 334–352 showed higher levels of CD62L and CD44 (~60%) as compared to controls, suggestive of activated/memory populations, and they were the major sources of cytokine production (Figure S5, panels left and right). It has been reported that Th1 cells producing IFN-γ and Th17 cells producing IL-17 family cytokines (mainly IL-17A) can induce myocarditis, but Th17 cells are critical for the development of dilated cardiomyopathy [38,39]. Similarly, Th cells producing GM-CSF (Th-GM cells) are reported to be proinflammatory in the induction of experimental autoimmune myocarditis [40] and other models [41,42,43]. Thus, our data supported the potential of Tg T cells to induce disease through the production of cytokines and indicate that the major cytokine producers might be CD4^+^ T cells since they account for 79% of T cells, as opposed to 13% of CD8^+^ T cells (Figure 1d). But because CD8^+^ T cells mediate their functions through cytotoxicity, it was critical to address this functionality.

**Figure 4:**
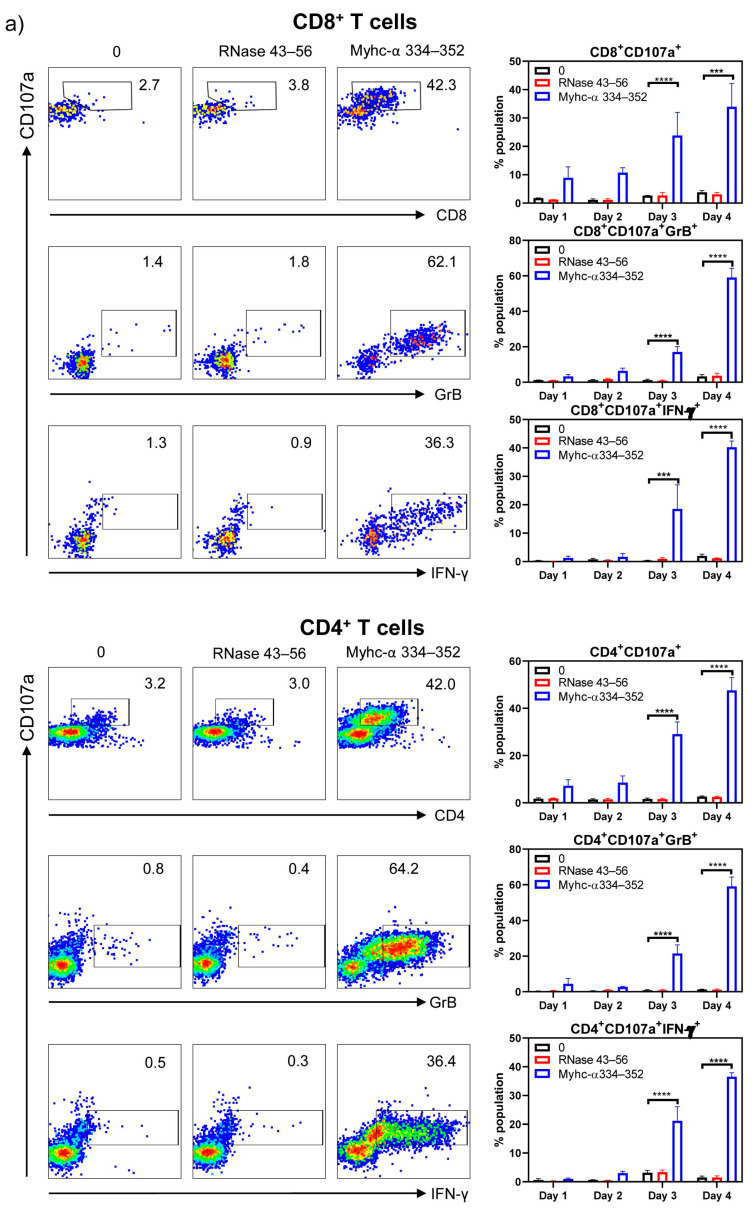

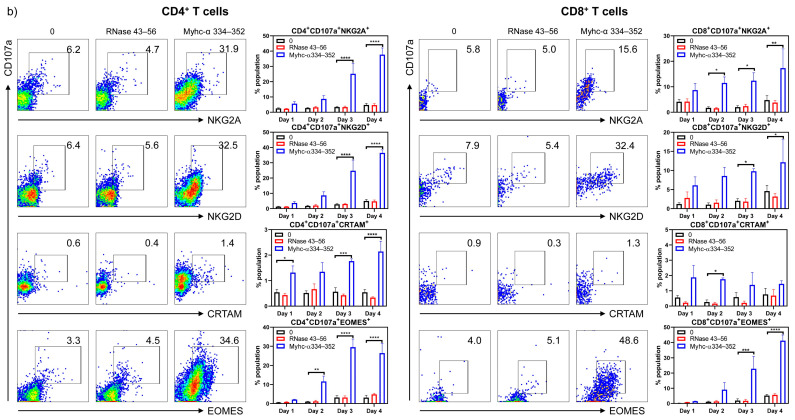
CD4^+^ T cells in Tg mice express markers of cytotoxicity similar to CD8^+^ CTLs. (**a**) Markers of CD8^+^ CTLs and their expression in CD4^+^ T cells. Lymphocytes obtained from naïve Tg mice were stimulated with or without Myhc-α 334–352 or RNase 43–56 (control) (50 µg/mL) for two days, and IL-2 medium was then added. On day 4 post-stimulation with peptides, surface expression of CD107a was analyzed with respect to CD8^+^ and CD4^+^ T cells. Additionally, intracellular staining was performed to analyze the expression of IFN-γ and GrB corresponding to CD8^+^ and CD4^+^ T cells. (**b**) Other markers of cytotoxic T cells. Cells were stained with antibodies for the indicated markers, and CD4^+^ or CD8^+^ T cells expressing CD107a were analyzed in relation to NKG2A, NKG2D, and CRTAM expression by flow cytometry. Intranuclear EOMES expression was evaluated after staining with antibodies for CD4 or CD8 and CD107a, as described in the Methods section. Mean ± SEM values are obtained from three mice in each group. * *p* ≤ 0.01, ** *p* ≤ 0.01, *** *p* ≤ 0.001, and **** *p* ≤ 0.0001.

To characterize the cytotoxic functions of CD8^+^ T cells, we examined the surface expression of CD107a and intracellular expression of GrB and IFN-γ, which are widely used as reliable markers of CD8^+^ CTLs, in response to antigenic stimulation [37,44,45,46,47,48,49]. We stimulated Tg T cells with or without Myhc-α 334–352 or RNase 43–56 (control) and analyzed their expression for four days post-stimulation by flow cytometry. As shown in Figure 4, top panel, CD107a was progressively upregulated in response to Myhc-α 334–352, and significant elevations were noted on days 3 to 4 (CD8^+^CD107a^+^ cells, 23.8 ± 8.2% to 33.9 ± 2.2%, *p* ≤ 0.001). By relating the CD107a expression to that of GrB and IFN-γ, it was clear that their expression overlapped (CD8^+^CD107a^+^GrB^+^, 17.1 ± 3% on day 3 vs. 59 ± 5.3% on day 4; and CD8^+^CD107a^+^IFN-γ^+^, 18.5 ± 8.4% on day 3 vs. 40.23 ± 2.2% on day 4, *p* ≤ 0.001). Since upregulation of CD107a, GrB, and IFN-γ occurred only in response to Myhc-α 334–352, the data suggested that CD8^+^ T cells responding to Myhc-α 334–352 possessed cytotoxic function.

However, since both CD4^+^ and CD8^+^ T cells responded to the same peptide (Myhc-α 334–352) in our transgenic model, we wished to examine the expression of cytotoxic markers in CD4^+^ T cells, expecting to find that they serve as a good control cell type for CD8^+^ T cells since CD4^+^ T cells are generally not expected to mediate cytotoxic functions. Contrary to our expectations, we noted that CD4^+^ T cells cultured in the presence of Myhc-α 334–352, but not controls, showed upregulation of CD107a (CD4^+^CD107a^+^, 29.1 ± 5.1% on day 3 vs. 47.6 ± 5.5% on day 4), GrB (CD4^+^CD107a^+^GrB^+^, 21.5 ± 4.9% on day 3 vs. 59 ± 5.3% on day 4), and IFN-γ (CD4^+^CD107a^+^IFN-γ^+^, 21.2 ± 4.9% on day 3 vs. 36.5 ± 1.4% on day 4) (Figure 4, bottom panel). Their patterns were similar to those observed in CD8^+^ T cells. While these findings supported the idea that CD4^+^ T cells responding to Myhc-α 334–352 may possess the characteristics of cytotoxic CD4^+^ T cells, we sought to further validate them by testing the expression of other newly described markers of CD4^+^ CTLs. These included surface expression of NKG2A [50], NKG2D [51], and CRTAM [52] and intracellular expression of EOMES [53,54,55]. In a flow cytometric analysis, we compared the expression of CD107a with that of NKG2A, NKG2D, CRTAM, and EOMES, expecting to find that their detection aligned with CD107a expression. The analyses revealed that upregulation of NKG2A and NKG2D occurred progressively, similar to CD107a expression, during days 3 to 4 in response only to Myhc-α 334–352 (CD4^+^CD107a^+^NKG2A^+^ or NKG2D^+^, 25 to 36%, *p* ≤ 0.001) (Figure 4b, left panel). Similarly, EOMES expression was increased from days 2 to 4 post-Myhc-α 334–352 stimulation (11.6 to 26.5%, *p* ≤ 0.01), whereas upregulation of CRTAM occurred relatively early on day 1, but its expression was low (1.7 to 2.1%, *p* ≤ 0.001) (Figure 4b, left panel). Reports indicate that expression of NKG2A and NKG2D signify inhibitory and activation functions, respectively, of CD4^+^ CTLs [50,56,57,58]. Since both molecules were upregulated corresponding to CD107a expression, our data did not support the proposition that NKG2A can be regarded as an inhibitory marker. While expression of EOMES agreed with previous observations [53], a marginal increase in the number of CRTAM^+^CD107a^+^ cells may suggest that CRTAM expression may not occur uniformly in CD4^+^ CTLs in response to different antigens. Of note, CRTAM was shown to be expressed between 6 and 24 h post-antigenic stimulation [59], but, in our hands, we did not detect its expression at the early time point. As for CD8^+^ T cells, unlike the conventional markers described above (CD107a, GrB, IFN-γ), NKG2A, NKG2D, CRTAM, and EOMES are also reported as markers of CD8^+^ CTLs in various systems [60,61,62,63,64,65]. Therefore, we tested their expression in our Tg model. Our data indicated that Myhc-α 334–352-responsive CD8^+^ T cells that express CD107a also showed upregulation of NKG2A (CD8^+^CD107a^+^NKG2A^+^, 11.5 to 17.3%, *p* ≤ 0.05), NKG2D (CD8^+^CD107a^+^NKG2D^+^, 9.8 to 12.1%, *p* ≤ 0.05), and EOMES (CD8^+^CD107a^+^EOMES^+^, 22.8 to 41.1%, *p* ≤ 0.001), whereas upregulation of CRTAM was low (~1.7%, *p* ≤ 0.05) (Figure 4b, right panel). Since expression patterns of NKG2A, NKG2D, and EOMES were similar between CD4^+^ and CD8^+^ T cells and also correlated with CD107a expression, the data suggested that they can be considered as markers of cytotoxicity of both CD4^+^ and CD8^+^ T cells. Alternatively, if activation facilitated upregulation of the above, then the CRTAM molecule should have been upregulated compared to the other markers indicated above, which was not the case. Thus, our data suggested that Tg Myhc-α 334–352-responsive CD4^+^ and CD8^+^ T cells could mediate tissue damage by multiple pathways that may involve the production of cytokines and cytotoxic effects.

**Figure 5:**
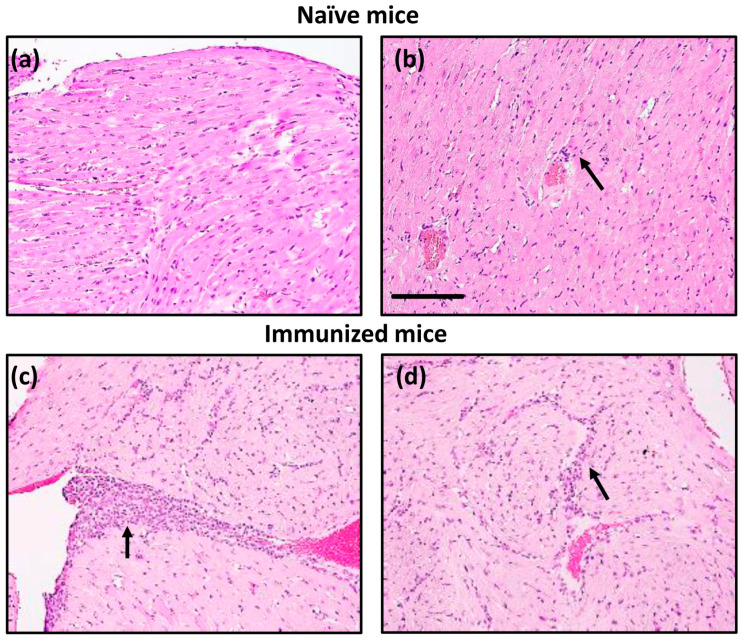
Tg mice develop myocarditis in response to Myhc-α 334–352 immunization. Naïve mice: Hearts collected from naïve Tg mice were evaluated for histological changes by H and E staining. Panels (**a**,**b**) indicate normal endocardium and minimal myocardial inflammation (arrow), respectively. Immunized mice: Tg mice were immunized with Myhc-α 334–352. On day 21, hearts were collected for histological assessment, as described above. Panels (**c**,**d**) indicate marked subendocardial myocarditis and myocardial mononuclear cell inflammatory infiltrates, respectively (arrows). Original magnification, ×20 (bar = 100 µm, applies to all panels). Representative images are shown from eight naïve and four immunized Tg mice.

By investigating the occurrence of myocarditis in naïve Tg mice during the ~4-month observation period, we noted that Tg mice were clinically normal. Histologically, their heart sections revealed only isolated lesions containing perivascular inflammatory cells in ~25% of animals (Figure 5, top panels). Whether aged animals develop more severe disease remains to be tested. Next, we explored whether immunization could hasten the development of myocarditis by administering CFA emulsions containing Myhc-α 334–352 [18,30]. Heart sections from immunized mice revealed multiple inflammatory foci suggestive of mild myocarditis (Figure 5, bottom panels). It is to be noted that we used fourth generation backcrossed mice to evaluate inflammatory changes. It is possible that further backcrossing mice to achieve the highest percentage of A/J genome, close to 99.99%, may develop a more severe myocarditis phenotype. However, it was essential to address the mechanisms underlying the development of myocarditis in immunized A/J Tg mice.

**Figure 6:**
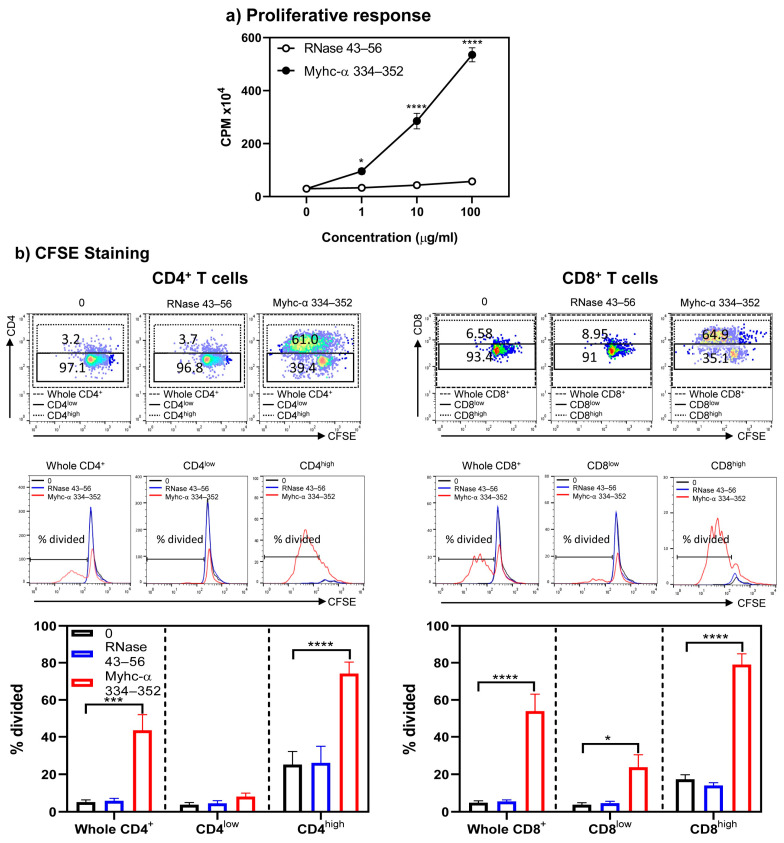
Proliferative responses of CD4^+^ and CD8^+^ T cells obtained from Tg mice immunized with Myhc-α 334–352 were comparable. (**a**) Proliferative response. Lymphocytes obtained from Tg mice immunized with Myhc-α 334–352 were stimulated with Myhc-α 334–352 or RNase 43–56 (control). After two days, cells were pulsed with [3H] thymidine, and 16 h later, proliferative responses were measured as CPM. (**b**) CFSE staining. Cells were stained with CFSE before stimulating with or without Myhc-α 334–352 or RNase 43–56 (50 µg/mL); IL-2 medium was added on day 2. On day 4, cells were stained with anti-CD4, anti-CD8, and 7-AAD. The top, middle, and bottom panels represent the flow cytometric plots of cells positive for CD4 and CD8 vs. CFSE; histogram plots of overlays between medium, RNase 43–56, and Myhc-α 334–352 stimulations; and bar diagrams of the whole CD4^+^ or CD8^+^ T cells and their respective low- or high-expressing cells. Mean ± SEM values are obtained from three mice in each group. * *p* ≤ 0.05, *** *p* ≤ 0.001, and **** *p* ≤ 0.0001.

In that direction, we first examined the proliferative responses of Tg T cells obtained from immunized mice. As expected, lymphocytes responded to Myhc-α 334–352 dose-dependently (*p* ≤ 0.05) compared to controls (medium and RNase 43–56) (Figure 6a). Notably, the threshold for Myhc-α 334–352 to stimulate Tg cells was low, as the lowest peptide concentration (1 µg/mL) could induce a 3-fold (*p* ≤ 0.05) increase in T cell responses. Such a response was lacking in naïve Tg mice (Figure 2a), suggesting that in vivo priming with peptides enhanced sensitivity to antigenic stimulation. We next compared differential proliferative responses, if any, between CD4^+^ and CD8^+^ T cells at a single-cell level by CFSE staining. The analyses revealed that CD4^+^ and CD8^+^ T cells showed upregulation of their corresponding co-receptors (Figure 6), as noted above, with naïve Tg T cells. The proliferating cells were found mainly in high populations of CD4^+^ or CD8^+^ cells in response to Myhc-α 334–352 (Figure 6, top and middle panels; *p* ≤ 0.001), and the percentages of divided cells were comparable between CD4^+^ and CD8^+^ T cell subsets (Figure 6, bottom panel).

**Figure 7:**
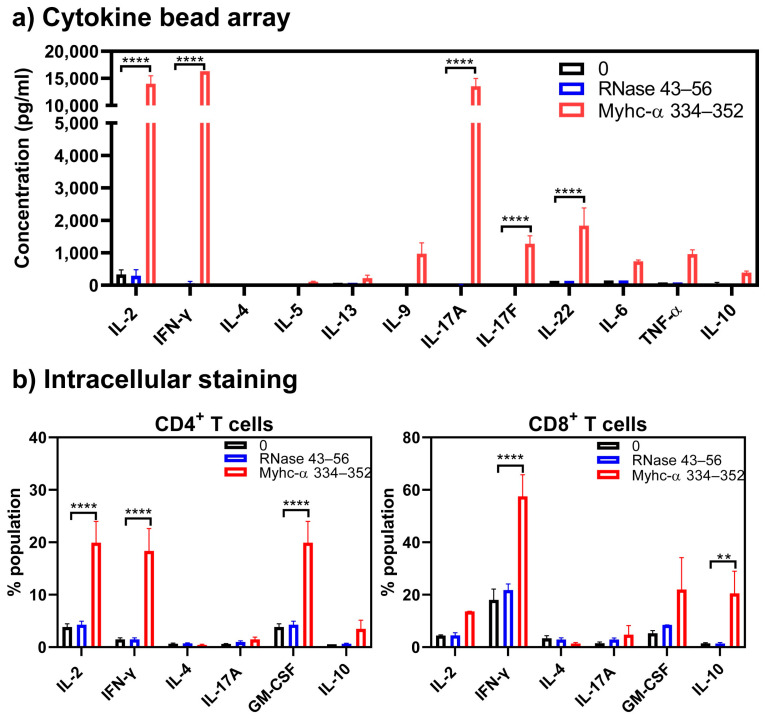
Tg T cells obtained from mice immunized with Myhc-α 334–352 produce mainly inflammatory cytokines. (**a**) Cytokine bead array analysis. Lymphocytes obtained from Tg mice immunized with Myhc-α 334–352 were stimulated with or without Myhc-α 334–352 or RNase 43–56 (control) (50 µg/mL); supernatants harvested on day 2 were analyzed for the indicated cytokines using the LEGENDplex Murine Th Cytokine Panel (12-plex; BioLegend), as described in the Methods section. (**b**) Intracellular staining. Cells were stimulated as above, and after two days, IL-2 medium was added. On day 4 post-stimulation, cells were stained with anti-CD4, anti-CD8, and 7-AAD. After fixation and permeabilization, cells were stained with antibodies for the indicated cytokines, and cells positive for each cytokine were analyzed in the CD4 or CD8 subsets by flow cytometry. Mean ± SEM values are obtained from three mice in each group. ** *p* ≤ 0.01 and **** *p* ≤ 0.0001.

Determination of cytokines by cytokine bead arrays revealed detection of mainly IL-2 (13,996.6 ± 1503.4 pg/mL, *p* ≤ 0.0001), IFN-γ (16,300 ± 0 pg/mL, *p* ≤ 0.0001), and Th17 cytokines—mainly IL-17A (13,539.1 ± 1443.8 pg/mL, *p* ≤ 0.0001) and, to a lesser degree, IL-17F (1277.5 ± 247.53 pg/mL, *p* ≤ 0.05) and IL-22 (1835 ± 548.8 pg/mL, *p* ≤ 0.01) (Figure 7a). At a single-cell level, we noted that the production of IL-2 (19.9 ± 4.1 pg/mL, *p* ≤ 0.0001), IFN-γ (18.4 ± 4.3 pg/mL, *p* ≤ 0.0001), and GM-CSF (19.9 ± 4.1 pg/mL, *p* ≤ 0.0001) was significantly high in CD4^+^ T cells stimulated with Myhc-α 334–352 (Figure 7b, left panel). While similar trends were noted with CD8^+^ T cells, differences were not significant with the frequency of GM-CSF^+^ cells. As for IL-10-producing cells, their frequency was high in the CD8^+^ T cell subset (20.0 ± 8.46%, *p* ≤ 0.01) but not in the CD4^+^ T cell subset (Figure 7b, right panel). Of note, based on the cytokine profiles determined by bead array analysis (Figure 7a), we expected the frequency of IL-17a^+^ cells to be high, but this was not the case. The discrepancy could be ascribed to the nature of the assays, since bead array analysis permits the measurement of cytokines accumulated over time as opposed to detection of cells producing cytokines at a specific time point via intracellular staining. Taken together, these findings suggested that administration of CFA/Myhc-α 334–352 enabled Tg T cells to produce Th17 in addition to Th1 cytokines, which might have facilitated the induction of myocarditis [39,66].

## 4. Conclusions

We recently reported the generation of TCR Tg mice specific to Myhc-α 334–352 on a myocarditis-resistant C57BL/6J genetic background [18]. Here, we have characterized Tg mice that were backcrossed onto an A/J genetic background for up to four generations and noted that both Tg CD4^+^ and CD8^+^ T cells from naïve mice expressing Myhc-α 334–352-specific TCRs specifically responded to the peptide antigen. While naïve Tg mice did not spontaneously develop myocarditis during the observation period of ~4 months, animals immunized with Myhc-α 334–352 developed mild myocarditis, which could be attributed to the production of Th1 and Th17 cytokines. Since CD8^+^ T cells expressed Myhc-α 334–352-specific TCRs similar to CD4^+^ Tg T cells, we provide evidence that Tg CD8^+^ T cells expressed the bona fide markers of cytotoxicity, CD107a, GrB, and IFN-γ. By extending these observations to CD4^+^ T cells, we unexpectedly noted that CD4^+^ T cells also expressed these same molecules in addition to NKG2A, NKG2D, and EOMES, signifying CD4^+^ CTLs [50,51,53,54] and indicating that both CD4^+^ and CD8^+^ Tg T cells produce comparable cytotoxic effects. Unlike other autoimmune disease models, the availability of Tg models to investigate the role of T cells in the development of cardiac autoimmunity is limited. Only one other TCR Tg model for Myhc 614–629 has been published to our knowledge, in which antigen-specific TCRs were found to be expressed only in CD4^+^ T cells [67]. Our Tg models available on both myocarditis-resistant (C57BL/6J) [18] and myocarditis-susceptible A/J genetic backgrounds (described here) may provide a useful framework to determine the role of T cells in genetic susceptibility to myocarditis. These aspects can be investigated in the context of both CD4^+^ and CD8^+^ T cells within a single transgenic system because both cell types express Myhc-α 334–352-specific TCRs. Additionally, our model provides opportunities to investigate the role of cytotoxic CD4^+^ T cells, as their role is beginning to be understood in various autoimmune settings [41,63,64,65,66,67]. Translationally, it is unknown whether CD4^+^ CTLs can contribute to cardiac damage in humans, but defining their antigen specificity may be challenging. Of note, immune suppressive therapies are generally used in myocarditis patients negative for viruses but positive for autoantibodies [68,69]. If antigen-specific T cells are identified as mediators of heart tissue destruction, the use of antigen-specific T cell therapies is not expected to interfere with the effects of immune suppressants.

## Data Availability

The original contributions presented in the study are included in the article/Supplementary Materials, further inquiries can be directed to the corresponding author.

## Supplementary Materials

The following supporting information can be downloaded at: https://www.mdpi.com/article/10.3390/cells13030234/s1. Figure S1: Memory CD4^+^ T cells and FoxP3-expressing CD4 T cells were relatively low in Tg mice. Figure S2: Cardiac myosin expression occurs in the heart but not in lymphoid organs. Figure S3: Tg T cells from naïve mice do not respond to the whole cardiac myosin protein. Figure S4: Lymphocytes from naïve Tg mice secrete mainly IFN-γ in response to anti-CD3 stimulation. Figure S5: Activated/memory CD4^+^ or CD8^+^ T cells expressing CD62L and CD44 were found to be the major sources of cytokine production.
