# Supplementary figures and images for: Investigation into Cardiac Myhc-α 334–352-Specific TCR Transgenic Mice Reveals a Role for Cytotoxic CD4 T Cells in the Development of Cardiac Autoimmunity
Source: Cells. 2024 Jan 26;13(3):234. doi: 10.3390/cells13030234 (PMC10854502; doi:10.3390/cells13030234)

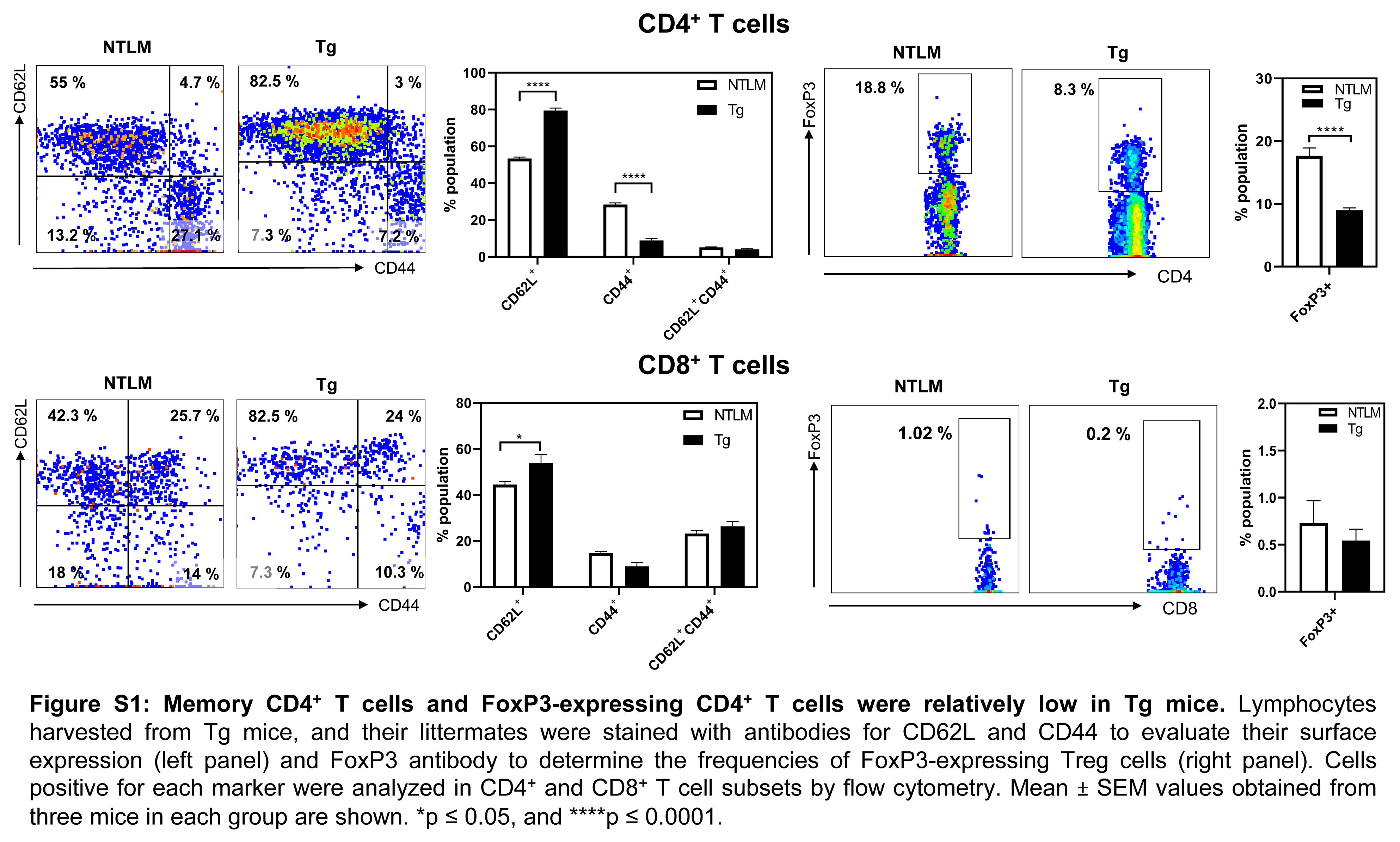

Supplement: Supplementary file 1 [file cells-13-00234-s001.zip › cells-2821469-supplementary/Figure S1 final.tif]

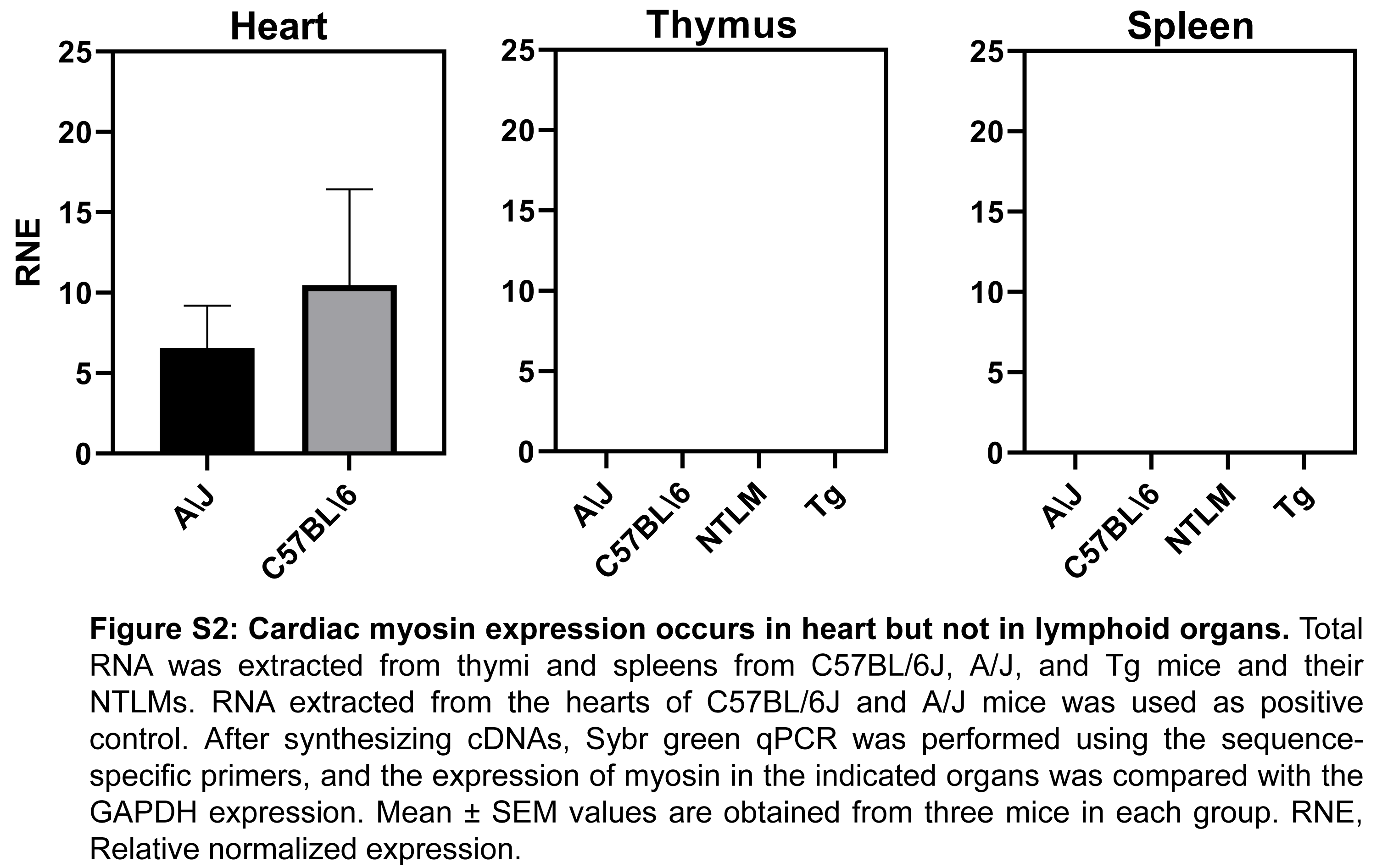

Supplement: Supplementary file 1 [file cells-13-00234-s001.zip › cells-2821469-supplementary/Figure S2 final.tif]

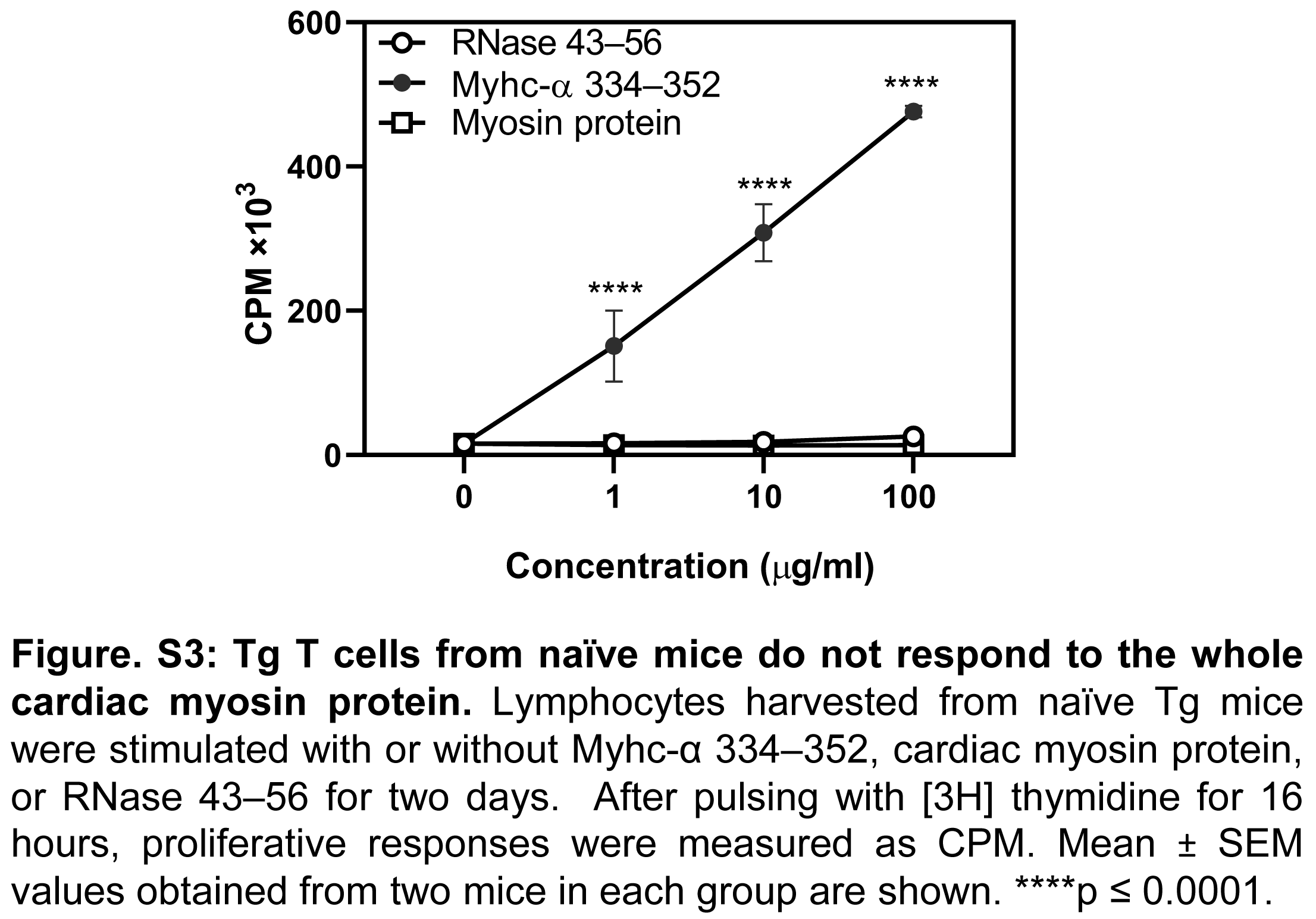

Supplement: Supplementary file 1 [file cells-13-00234-s001.zip › cells-2821469-supplementary/Figure S3 final.tif]

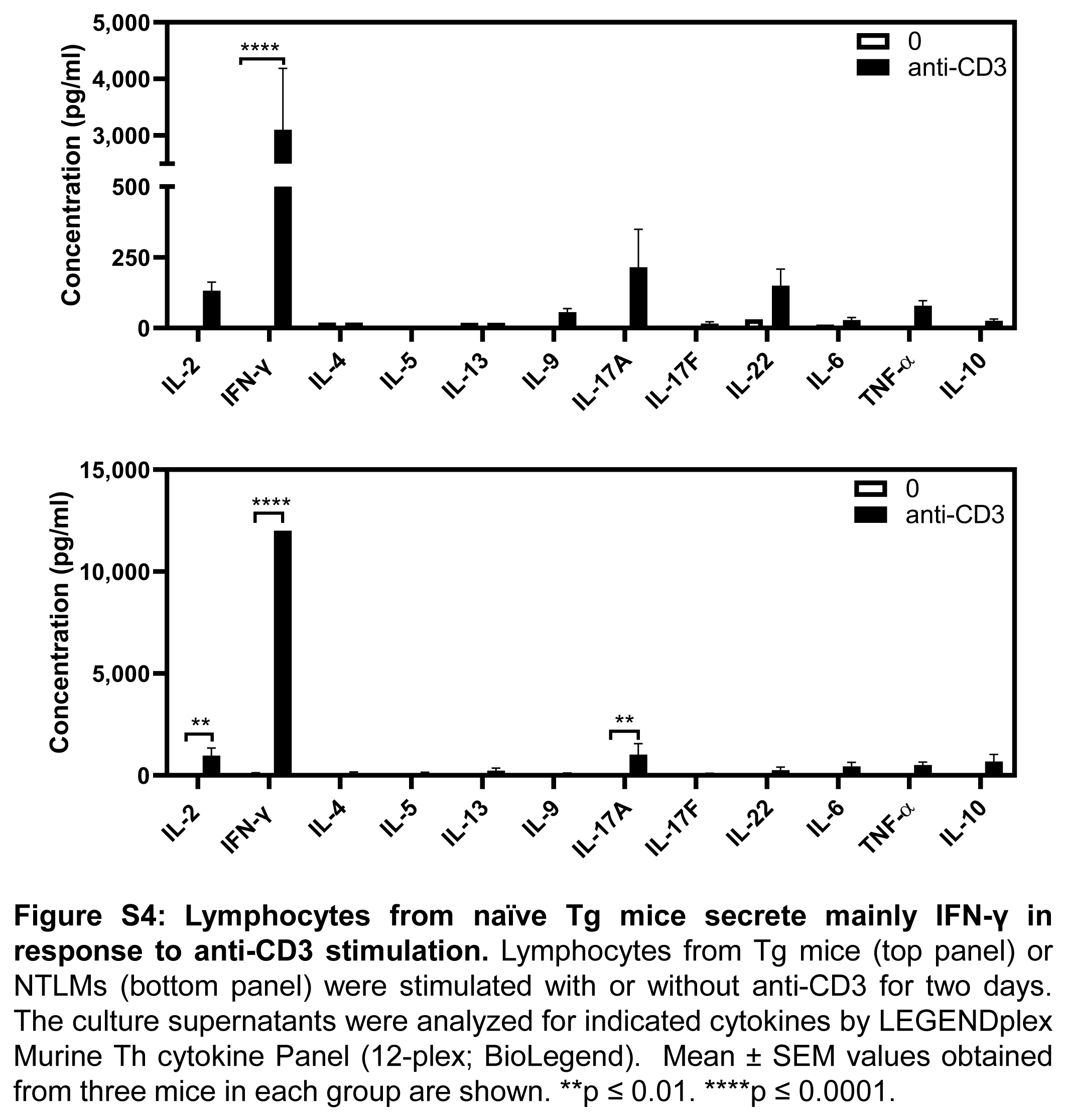

Supplement: Supplementary file 1 [file cells-13-00234-s001.zip › cells-2821469-supplementary/Figure S4 final.tif]

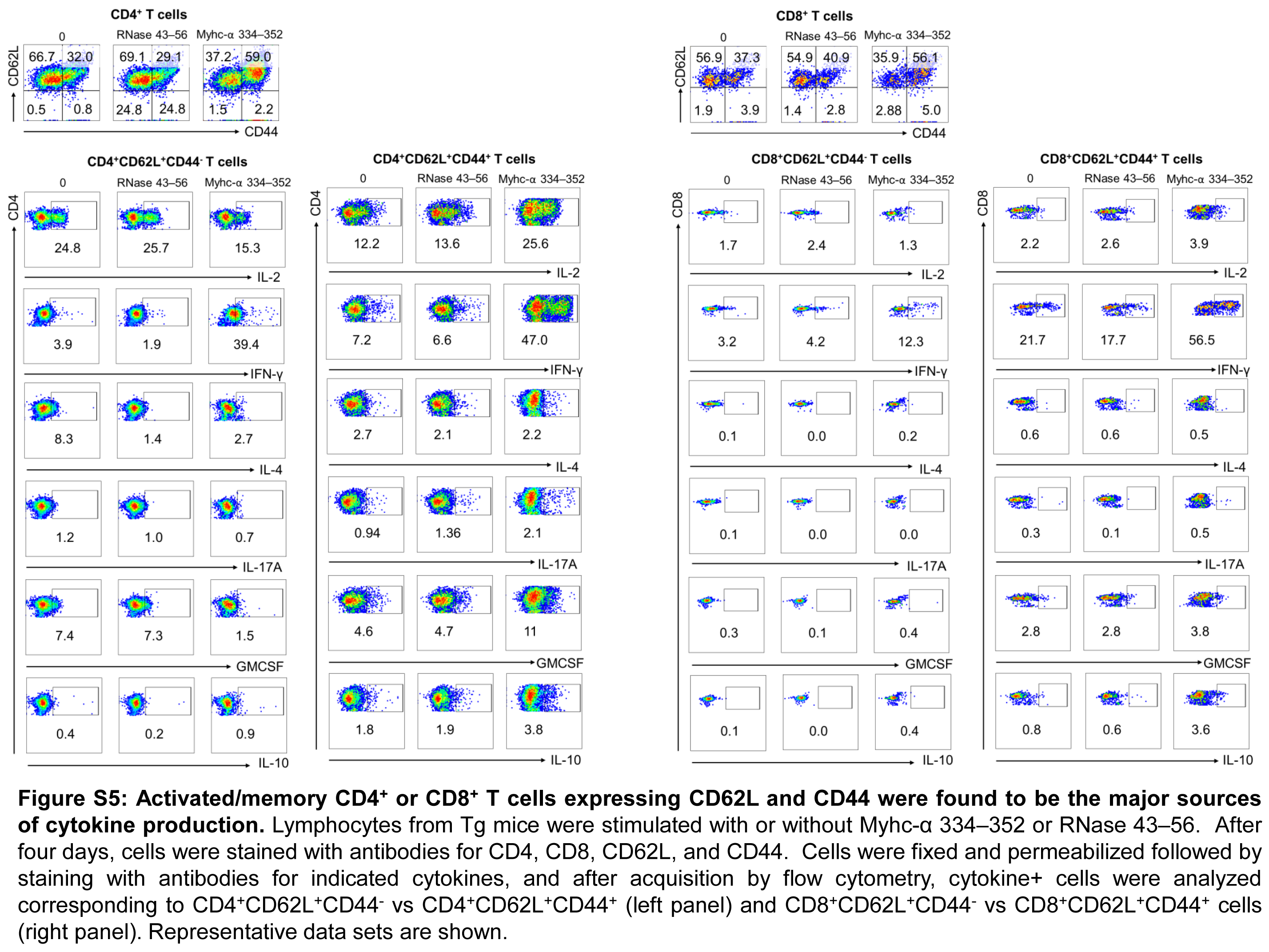

Supplement: Supplementary file 1 [file cells-13-00234-s001.zip › cells-2821469-supplementary/Figure S5 final.tif]
